# Genotype-phenotype correlations in *PMS2*-associated constitutional mismatch repair deficiency: a systematic literature review

**DOI:** 10.3389/or.2025.1679576

**Published:** 2025-11-17

**Authors:** Cătălin Vasile Munteanu, Diana Luisa Lighezan, Alexandru Capcelea, Adela Chiriță-Emandi, Adrian Pavel Trifa

**Affiliations:** 1 Doctoral School, Victor Babeş University of Medicine and Pharmacy, Timişoara, Romania; 2 Regional Center of Medical Genetics Timiş, Louis Țurcanu Clinical Emergency Hospital for Children, Timişoara, Romania; 3 Department of Hematology, Victor Babes University of Medicine and Pharmacy, Timişoara, Romania; 4 Multidisciplinary Research Center for Malignant Hematological Diseases, Victor Babes University of Medicine and Pharmacy, Timişoara, Romania; 5 Department of Medical Oncology, OncoHelp Oncology Center, Timişoara, Romania; 6 Department of Microscopic Morphology, Genetics Discipline, Victor Babeş University of Medicine and Pharmacy, Timişoara, Romania; 7 Center for Genomic Medicine, Victor Babeş University of Medicine and Pharmacy, Timişoara, Romania; 8 Center of Expertise on Rare Pulmonary Diseases, Victor Babeş Clinical Hospital of Infectious Diseases and Pneumophysiology, Timişoara, Romania; 9 Breast Cancer Center, The Oncology Institute “Prof. Dr. Ion Chiricuta”, Cluj-Napoca, Romania

**Keywords:** constitutional mismatch repair deficiency, *PMS2*, genotype, Lynch, VarChat

## Abstract

Constitutional mismatch repair deficiency (CMMRD) is a rare pediatric cancer predisposition syndrome primarily characterised by central nervous system (CNS), gastro-intestinal (GI) tumours and hematological malignancies, along with NF1-like cutaneous features. The *PMS2*-related subtype (*PMS2*-CMMRD) is the most common molecular form of CMMRD, exhibiting variable severity and both early and late-onset clinical presentations. Although pathogenic and likely pathogenic *PMS2* heterozygous variants are relatively frequent in healthy population, CMMRD incidence is generally rare in humans and genotype-phenotype correlations are still limited. To better characterise *PMS2*-CMMRD group, we collected clinical cases described in literature, using three alternative methods (VarChat, VarSome and LitVar2), starting from 102 pathogenic/likely pathogenic *PMS2* variants (<50 bp) reported in ClinVar by clinical and research laboratories. *PMS2*-CMMRD cases were split into two distinct groups based on tumour onset age: early (diagnosis under 10 years) and later-onset (diagnosis after 10 years). Significant differences in tumour distribution were observed, with CNS tumours being most prevalent in the early-onset group, while GI tumours were more common in the later-onset group. Six *PMS2* variants were associated with either early or later-onset CMMRD. Future validation through larger prospective cohort studies is necessary to confirm our findings and better understand the natural history of *PMS2*-CMMRD to inform clinical decision-making in *PMS2*-Lynch syndrome (*PMS2*-LS).

## Introduction

Constitutional mismatch repair deficiency (CMMRD) syndrome (OMIM #276300, #619096, #619097, #619101) is a rare autosomal recessive cancer predisposition syndrome manifesting in childhood, associated with biallelic germline variants in mismatch repair (MMR) genes, *MLH1, MSH2, MSH6* and *PMS2*. Affected individuals typically develop early-onset malignancies, with central nervous system, hematological and gastro-intestinal tumours being the most prevalent neoplasias in this group (1–3). As clinical phenotype in CMMRD overlaps with other rare genetic diseases, such as neurofibromatosis type 1 (NF1) and Legius syndrome (4–6), timely diagnosis plays an essential role for appropriate clinical care and genetic counselling.

Among reported CMMRD cases, those associated with biallelic *PMS2* variants are the most prevalent in literature, compared to presentations involving other Lynch syndrome-associated MMR genes (7–9). In contrast, heterozygous *PMS2* variants are typically associated with lower penetrance and later-onset disease, with *PMS2*-associated Lynch syndrome (*PMS2*-LS) considered the mildest and the most frequently underdiagnosed form of LS documented to date (8,10–12). However, genotype–phenotype correlations in both *PMS2*-CMMRD and *PMS2*-LS remain poorly defined. Despite the presumed high prevalence of pathogenic *PMS2* variants in the general population, clinical data on disease progression in relation to specific genotypes remain scarce. For other similar recessive cancer predisposition syndromes, including Fanconi anemia (FA), emerging genotypic data are demonstrating the role of specific variants in disease development (13–15). These insights not only impact the clinical management of biallelic carriers, but also provide valuable data regarding heterozygous carriers of low penetrance variants associated with milder cancer predisposition phenotypes, contributing to more accurate risk assessment and enabling personalized follow-up strategies distinct from conventional gene-based approaches (15–17).

In this context, we aimed to systematically investigate *PMS2*-related CMMRD cases documented in scientific literature to date and reported in ClinVar, the most widely used clinical genomic database worldwide. The primary source of data was represented, in the vast majority of instances, by case reports and case series from which both clinical and molecular information were extracted. In our endeavour, we primarily focused on detailed genotype and phenotype characterisation of the cases under analysis, as well as on discovering potential genotype-phenotype correlations relevant for clinical practice.

## Methods

### Variant selection

All *PMS2* variants submitted by clinical and research laboratories to ClinVar were analysed, with ClinVar serving as the genomic database for this study (last accessed 1 May 2025). Only short variants (<50 bp) classified as pathogenic (class 5) and likely pathogenic (class 4) were included. Clinical significance for all variants were established according to the ACMG/AMP 2015 guidelines (18) by independent clinical and research laboratories or expert panels (19), with all variants meeting the ClinVar one-star criteria at least. Seven variants with conflicting interpretations (uncertain significance versus pathogenic/likely pathogenic) were not considered. Variants associated with constitutional mismatch repair deficiency (CMMRD) were selected based on the presence of one of the following terms in ClinVar records: “CMMRD,” “constitutional,” “homozygous” and “compound heterozygous.”

### Clinical cases discovery

**FIGURE 1:**
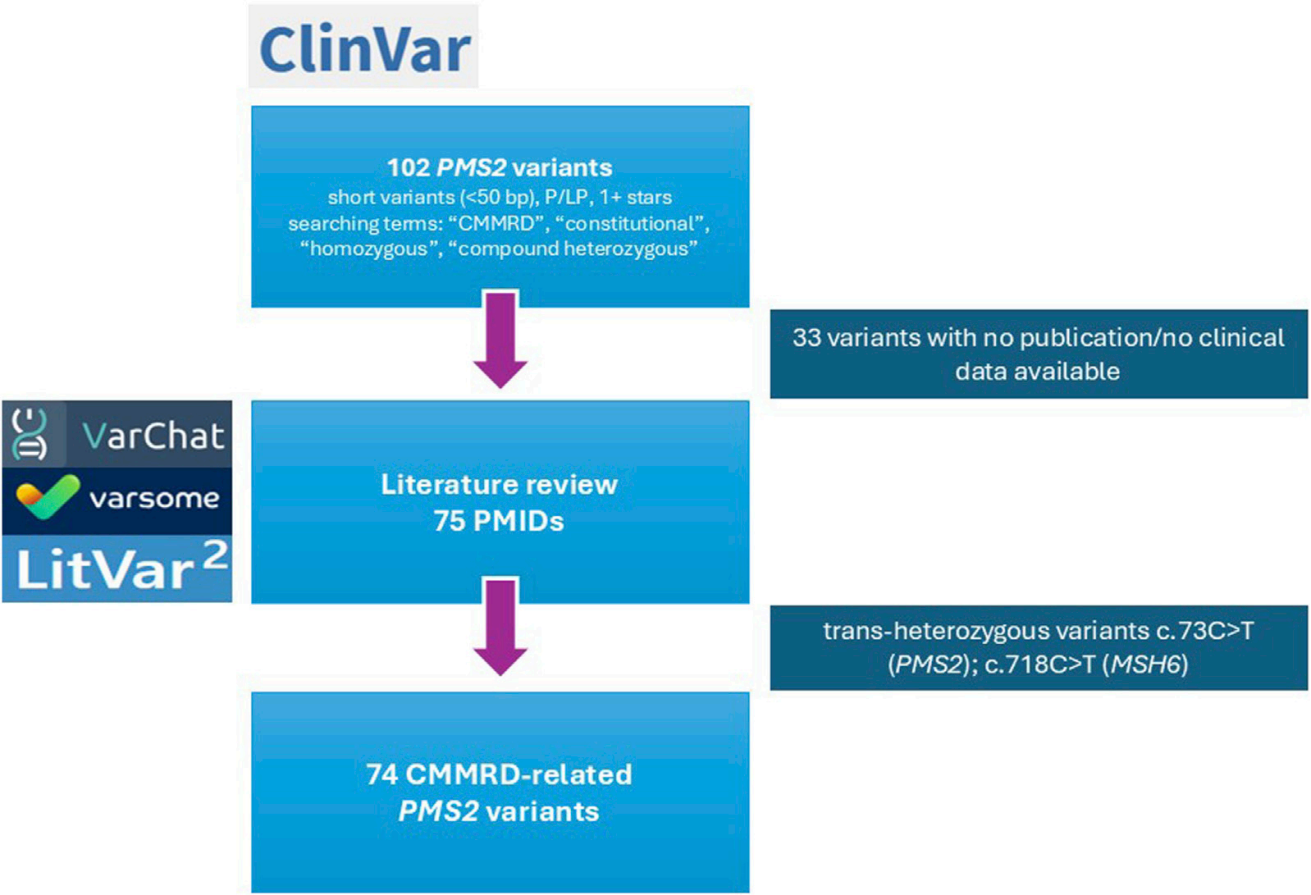
Flowchart of CMMRD-related *PMS2* variant selection. The process involved initial variant retrieval from ClinVar, literature review using VarChat, VarSome and LitVar2 and final manual curation. Excluded variants (n = 33) were subjected to an additional manual review.

After variant selection, 102 *PMS2* variants were further evaluated for supporting publications in the scientific literature. *PMS2* variants were annotated following the Human Genome Variation Society (HGVS) nomenclature guidelines (https://hgvs-nomenclature.org/stable/), using the MANE Select transcript (NM_000535.7; ENST00000265849.12) as reference, where c.1 denotes the first coding nucleotide. The literature review was conducted in a semi-automated manner based on the HGVS nomenclature of each variant, using three alternative tools: VarSome (https://varsome.com/) (20), LitVar2 (https://www.ncbi.nlm.nih.gov/research/litvar2/) (21) and VarChat (https://varchat.engenome.com/) (22). Moreover, citations supporting the germline classification of variants in ClinVar, as provided by other submitting laboratories, were manually reviewed. For variants with no publications identified using mentioned resources, an additional manual literature review in PubMed was performed by two independent researchers to ensure a comprehensive analysis (Figure 1).

Variants with no literature evidence supporting an association with CMMRD were excluded, resulting in an initial list of 69 *PMS2* variants. Following literature review, the number of short variants (<50 bp) increased to 74 and 8 exonic copy number variants (CNVs, >50 bp) were found in *trans* with the original variants (Figure 1).

Based on 75 PubMed indexed articles (2, 6, 10), (23–40), (41–60), (61–80), (81–94), we build a clinical database with 133 entries comprising patients and/or families with CMMRD. Two cases were excluded: 1) one case with the genotype c.[746_753del]; [1738A>T] and clinical presentation not suggestive of CMMRD (colorectal cancer at age 69) (31), 2) one case with trans-heterozygosity for c.73C>T (*PMS2*) and c.718C>T (*MSH6*), presenting with CMMRD features (glioblastoma at age 8 and café-au-lait macules) (27). Five cases with only one pathogenic *PMS2* variant reported but with clinical features suggestive of CMMRD were included, under the assumption that the second variant might have gone undetected due to technical limitations. A small number of CMMRD cases incidentally identified during the literature review, for which the genotypes were not reported in ClinVar, were not further assessed.

We constructed an internal review database comprising 133 entries of individuals and families with constitutional mismatch repair deficiency (CMMRD). In the vast majority of cases, each entry represents an individual. However, for three entries, the data reflect families rather than single individuals, due to insufficient clinical details in the original publications to distinguish separate cases. When publications provided enough clinical data and genotype inference was possible, typically for individuals identified through cascade testing or those with very suggestive phenotypes, they were included separately, even if not specifically mentioned in the original papers.

### Control variants

The 74 variants to study were compared with a control group consisting of 733 *PMS2* short variants (<50 bp) concordantly classified as pathogenic (class 5) or likely pathogenic (class 4) in all ClinVar submissions but not associated with CMMRD. Control variants were identified by excluding any variants retrieved using the CMMRD-related keywords described in the variant selection section. Variants that were initially considered for the study group but subsequently excluded were not included in the control group.

### Variant annotation and statistical analysis

Both study variants and control variants were annotated using GeneBe (https://genebe.net/) (95) and Ensembl Variant Effect Predictor (VEP) (https://ensembl.org/Homo_sapiens/Tools/VEP/). The reference human genome used was GRCh38. Variant nomenclature followed the Human Genetic Variation Society guidelines (https://hgvs-nomenclature.org/stable/). The MANE Select transcript (NM_000535.7, ENST00000265849.12) represented the reference sequence, with position c.1 being the first coding nucleotide. Splicing impact was predicted *in silico* using three complementary tools, SpliceAI (https://spliceailookup.broadinstitute.org/) (96), SpliceAI-visual (https://mobidetails.chu-montpellier.fr/) (97) and SPiCEv2.1 (98). Statistical analysis was performed using IBM SPSS Statistics 27. Statistical significance was defined for p-values <0.05.

## Results

### Targeted gene testing–the major approach for establishing definitive molecular diagnostic in CMMRD

For the majority of cases, 73/133 (53.2%), the first-tier molecular testing available was targeted *PMS2* gene testing, typically guided by initial immunohistochemistry (IHC) results. This included DNA sequence analysis (based on long-range PCR, Sanger sequencing and MLPA), RNA sequencing (based on RT-PCR and Sanger sequencing) and combined testing (both DNA and RNA). In 14/133 cases (10.2%), NGS panels were the preferred diagnostic tool. Exome sequencing (both standard and enhanced versions) was used in 10/133 cases (7.3%), while genome sequencing was employed in 3/133 cases (2.1%). In 37/133 cases (27.0%), the preferred testing approach could not be definitively determined.

### Brain tumours followed by gastro-intestinal tumours represent the most common sequence in the natural history of *PMS2*-CMMRD

**FIGURE 2:**
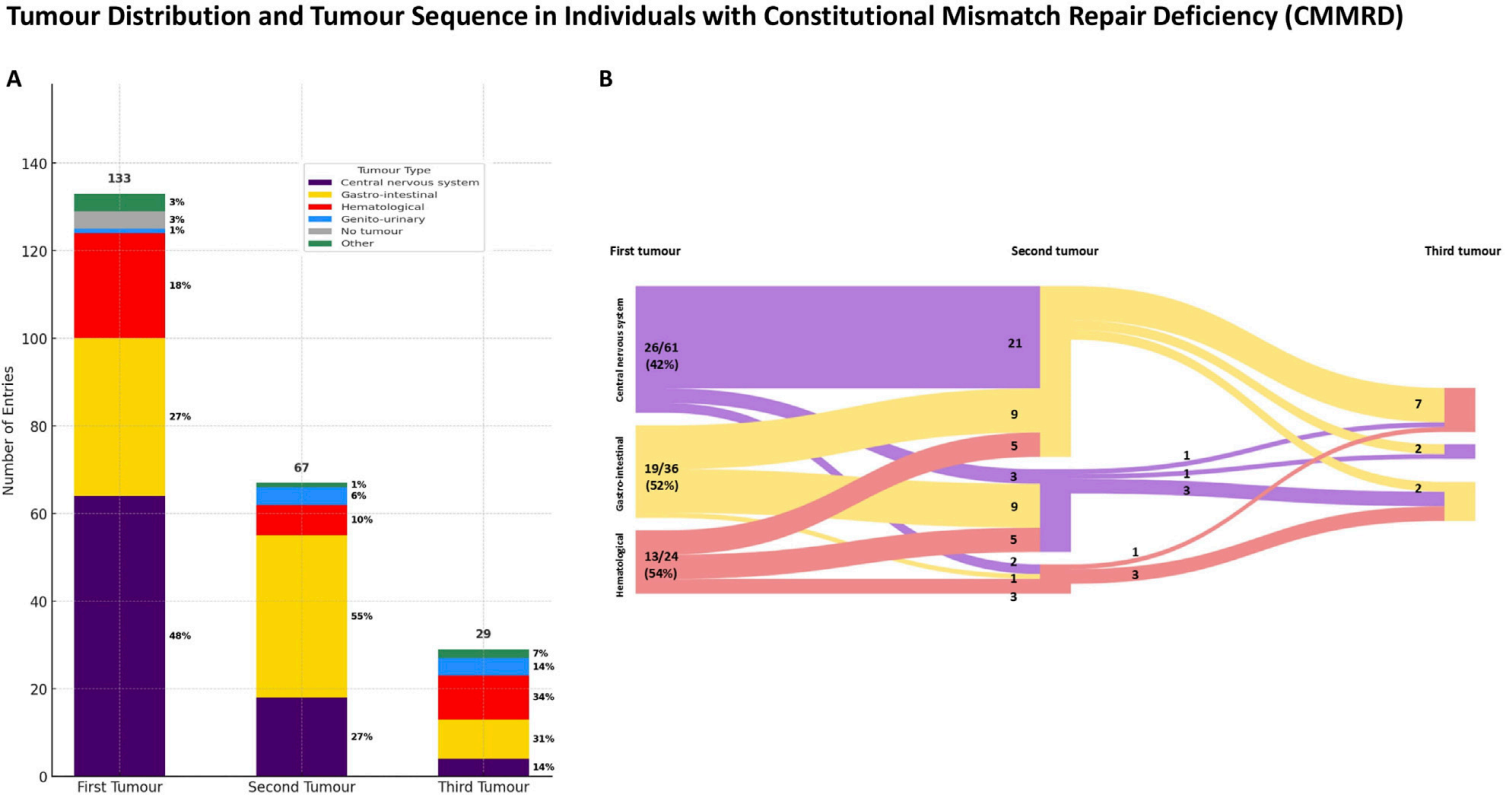
Tumour distribution **(A)** and tumour sequence **(B)** in individuals with *PMS2-*reated constitutional mismatch repair deficiency (*PMS2-*CMMRD). **(A)** Note that central nervous system tumours were the most prevalent as an initial presentation, followed by gastro-intestinal malignancies that occurred more frequently subsequently during the disease course. **(B)** Diagram B displays only reported cases with two or more tumours, with both absolute and relative numbers shown. Percentages in diagram B represent the proportion of cases presenting at least two neoplasms. For simplicity, cases with other tumour types were excluded.

In 129/133 (97%) of cases, individuals with CMMRD developed at least one tumour (Figure 2), with central nervous system tumours being the most common neoplasia in the natural history (p < 0.001, χ^2^). In 67/133 (50.3%) of cases, a second tumour occurred, most commonly in the gastro-intestinal tract. Only 29/133 (21.8%) of cases developed a third tumour during the disease course, with hematological (10/29, 34%) and gastro-intestinal (9/29, 31%) tumours being the most common.

### Brain tumours are the most frequent first neoplasia in early-onset *PMS2*-CMMRD, while gastro-intestinal tumours predominate in later-onset cases

**FIGURE 3:**
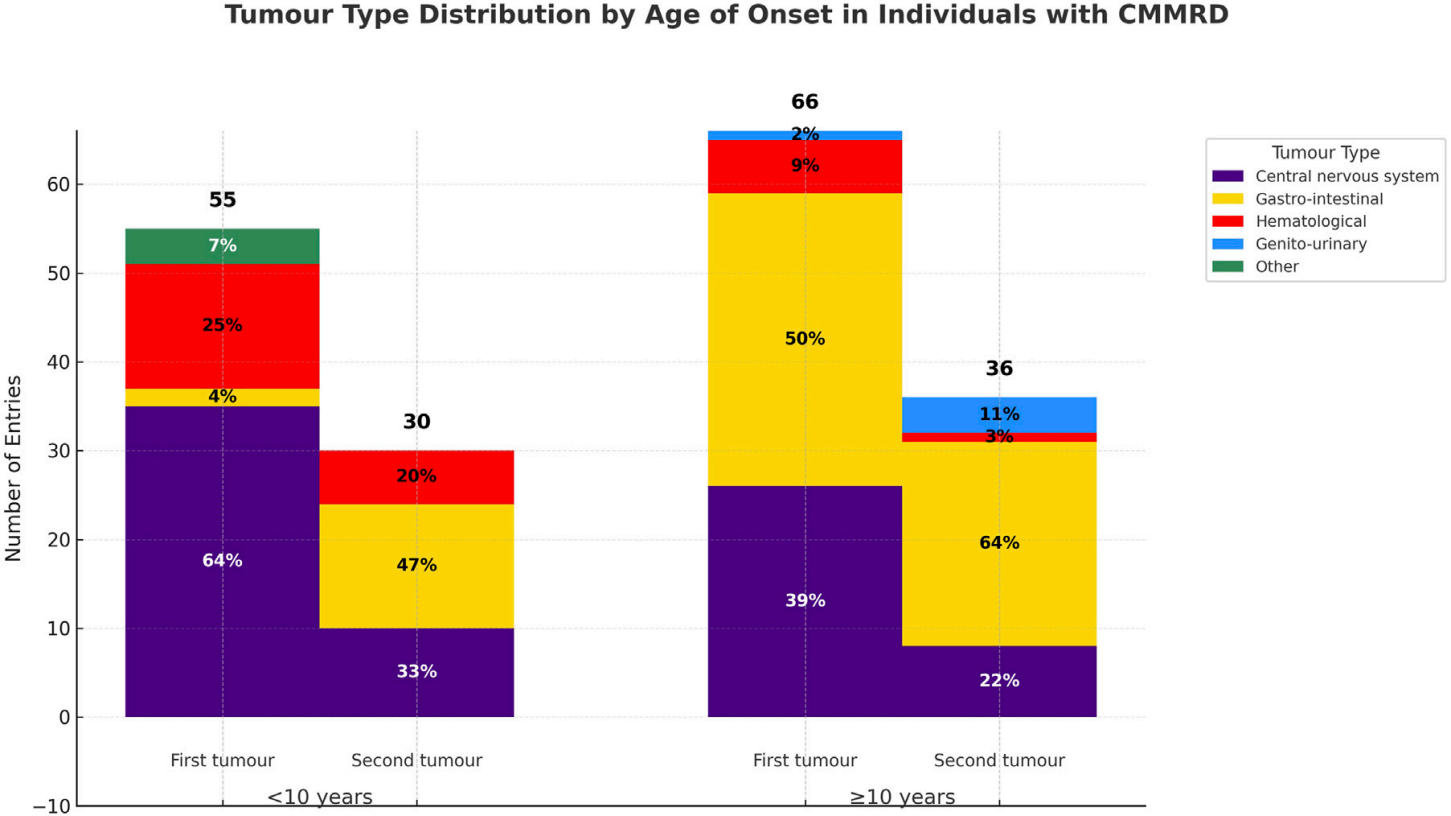
Tumour distribution in early and later-onset *PMS2-*CMMRD cases. Central nervous system tumours were the most common initial malignancy in early-onset cases, while gastro-intestinal tumours predominated as a first presentation in later-onset cases. Gastro-interstinal tumours were the major presentation in the evolution of both groups.

First neoplasia developed before age 10 in 55/129 (42.6%) of cases, whereas 66/129 (51.1%) had a later onset (Figure 3). Notably, gastro-intestinal tumours were rare as first presentations in early-onset (<10 years) CMMRD (p < 0.001, χ^2^), but represented 50% of initial tumours in later-onset cases (≥10 years). For 8/133 (6%) of cases, the age of onset could not be determined and the remaining 4/133 (3%) were tumour-free at the moment of reporting. While observed in both groups, the relative reduction in the number of brain tumours (p = 0.007, χ^2^) and the increase in the proportion of gastro-intestinal tumours (p < 0.001, χ^2^) from the first to the second neoplasia were significant only in the early-onset group. Even though haematological tumours were not the most common first presentation, they occurred earlier at a median age of 6 years, whereas CNS tumours were diagnosed at a median age of 7.5 years; nevertheless, the difference was not statistically significant (p = 0.41, Mann–Whitney U test).

### Rare tumoural and other non-tumoural phenotypes in *PMS2*-associated constitutional mismatch repair deficiency

Apart from early-onset central nervous tumours, gastro-intestinal and hematological neoplasms, the CMMRD cases included in this study presented with other clinical features, mainly dermatological, and less commonly reported immunological (3/133, 2.25%) and neuropsychiatric (5/133, 3.75%) manifestations. Rare neoplasms were also reported in several cases (Table 1).

**TABLE 1:** Summary of cutaneous manifestations and rare tumours in CMMRD cases. NF1-like phenotype was the most frequently reported clinical non-tumoural presentation in the CMMRD group. Both absolute and relative counts are provided for each clinical feature.

Dermatological signs	Cases	(%)	Rare tumours	Cases	(%)
NF1 “features” (CALMs and/or freckling)	65	48.87	Gastric cancer	2	1.50
Hypopigmented macules	6	4.51	Osteosarcoma	2	1.50
Cutaneous nevi	5	3.75	Brain angioma	1	0.75
Pilomatricomas	3	2.25	Basal cell carcinoma	1	0.75
Hemangiomas/vascular malformations	2	1.50	Cerebral angiosarcoma	1	0.75
Adenoma sebaceum	1	0.75	Dermatofibrosarcoma protuberans	1	0.75
Blaschkoid hyperpigmentation	1	0.75	Endometrial cancer (clear cell)	1	0.75
Dermoid cyst	1	0.75	Infantile myofibromatosis	1	0.75
Lichen planus	1	0.75	Melanoma	1	0.75
			Optic pathway glioma	1	0.75
			Rhabdomyosarcoma	1	0.75

### Genotypic characteristics of the study group

**FIGURE 4:**
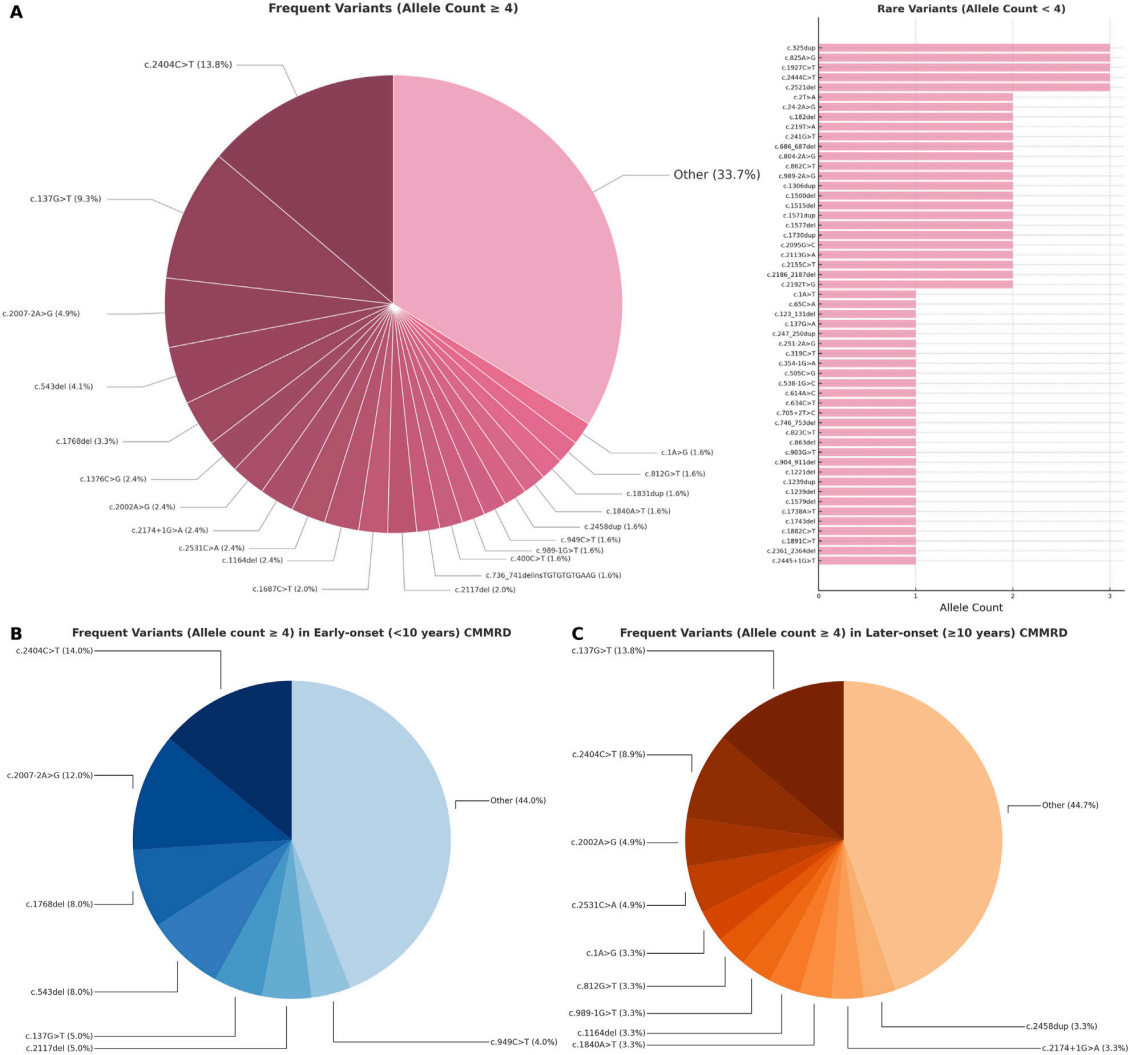
Distribution of short *PMS2* variants (<50 bp) in CMMRD cases: **(A)** all reported cases, **(B)** early-onset and **(C)** later-onset presentations. Variants are shown proportionally, according to allelic frequency in the review database.

We identified 74 short *PMS2* variants associated with CMMRD cases that were reported in ClinVar (Figure 4). Among them, 28/74 (37.83%) were frameshift variants, 18/74 (24.32%) stopgain variants (including both nonsense and frameshifts variants creating stop codons at the same genomic site), 13/74 (17.56%) missense, 11/74 (14.86%) splicing (excluding missense and frameshifts located at canonical splice sites), 3/74 (4.05%) startloss and 1/74 (1.35%) synonymous. Homozygosity was noted in 79/133 (59.39%) of entries and 33/74 (44.59%) of variants, while 54/133 (40.60%) of entries and 48/74 (64.86%) of variants were compound heterozygous. The large proportion of homozygous cases coincided with a large number of homozygous variants reported in consanguineous families (16 out of 36 homozygous variants, 44.44%) and founder/recurrent variants (10 out of 36 homozygous variants, 27.77%). In 15/133 (11.12%) of entries, compound heterozygosity involved exonic copy number variants (deletions or duplications). Genotypes weighted by their frequency in our database are illustrated in Figure 4.

### Later-onset *PMS2*-CMMRD cases are enriched in splicing variants with mild predicted impact

Of the variants reported in cases with onset after 10 years of age, 12/48 (25%) had a predicted splicing impact, whereas in cases with onset before 10 years, 7/39 (17.94%) involved splicing variants. In the later-onset group (≥10 years), in-frame exon skipping and leaky splicing events were more frequently predicted (Table 2). Non-spliceogenic truncating variants were a more frequent occurrence in the early-onset group. However, in the later-onset cases, most variants were clustered in exon 11 of the gene.

**TABLE 2:** Comparison between predicted spliceogenic effects of *PMS2* variants identified in early-onset (<10 years) and later-onset (≥10 years) CMMRD groups. *In silico* predictions were generated using SpliceAI, SpliceAI-visual, SPiCE and the Ensembl Variant Effect Predictor (VEP).

Early-onset CMMRD	*In silico* predictions/functional impact	Zygosity/second allele	References	Later-onset CMMRD	*In silico* predictions/functional impact	Zygosity/second allele	References
Splicing impact	7/39 (17.94%)				12/48 (25%)		
c.325dup	Cryptic donor, frameshift, mutant leaky splicing	Compound heterozygous/?	(76)	c.24-2A>G	Cryptic acceptor, frameshift, NMD escaping variant	Homozygous	(61)
c.538-1G>C	Cryptic acceptor, frameshift	Compound heterozygous/exons 6_8 deletion	(62)	c.251-2A>G	Frameshift, wild-type leaky exon skipping	Compound heterozygous/c.1A>G	(10)
c.825A>G	Synonymous, cryptic acceptor, frameshift	Compound heterozygous/c.2444C>T	(94)	c.325dup	Cryptic donor, frameshift, mutant leaky splicing	Compound heterozygous/c.825A>G	(2)
c.903G>T	Missense, frameshift	Compound heterozygous/exon 11_12 duplication	(28, 57)	c.354-1G>A	Wild-type and mutant intronic inclusion	Compound heterozygous/c.137G>T	(57)
c.904_911del	Cryptic acceptor, frameshift	Compound heterozygous/c.1882C>T	(23)	c.705+2T>C	In-frame exon skipping	Compound heterozygous/?	(64, 75)
c.2007-2A>G	Cryptic acceptor, frameshift, in-frame exon skipping	Homozygous	(39, 57)	c.804-2A>G	Frameshift, wild-type leaky exon skipping	Compound heterozygous/c.137G>T	(28, 77)
c.2174+1G>A	In-frame exon skipping	Homozygous	(70, 72)	c.812G>T	Missense, frameshift, wild-type and mutant leaky exon skipping	Homozygous	(84)
				c.825A>G	Synonymous, cryptic acceptor, frameshift	Compound heterozygous/c.325dup	(2)
				c.989-2A>G	In-frame exon skipping	Homozygous	(24)
				c.989-1G>T	In-frame exon skipping	Homozygous	(78)
				c.2002A>G	Missense, cryptic donor, frameshift, leaky splicing	Homozygous	(26, 36, 60)
				c.2174+1G>A	In-frame exon skipping	Homozygous	(70, 72)
Missense	5/39 (12.82%)				8/48 (16.66%)		
Frameshift/nonsense	28/39 (71.79%)				27/48 (56.25%)		
	10/28 (35.71%) in exon 11				16/27 (59.25%) in exon 11		

Allele unknown, NMD, nonsense-mediated decay.

### *PMS2* variants associated with early-onset (<10 years) and later-onset (≥10 years) CMMRD

To minimize the impact of shared polygenic background and non-genetic environmental exposures that may additionally contribute to phenotypic similarity among family members, we filtered out *PMS2* variants reported exclusively within a single family. Due to the limited number of case reports in the review database, variants observed in at least three unrelated individuals were considered sufficiently significant for further analysis. Moreover, inclusion was restricted to variants with an allele count greater than four, thereby excluding those documented solely in two homozygous individuals. Variants occurring exclusively in individuals with CMMRD presenting with early (<10 years) or later (≥10 years) onset were reported in Table 3. Two *PMS2* variants were exclusively identified in early-onset CMMRD cases (c.2007-2A>G and c.2117del), while four variants were uniquely observed in later-onset cases (c.1A>G, c.2002A>G, c.2458dup and c.2531C>A).

**TABLE 3:** *PMS2* variants exclusively reported in early (<10 years) and later-onset (≥10 years) CMMRD groups.

*PMS2* variant	Predicted consequence	Germline, heterozygous variant (literature)	gnomAD v4.1.0 allelic frequency	CMMRD cases (review database)	Comments
Early-onset (<10 years) CMMRD group					
c.2007-2A>G	Splice acceptor variant	Colorectal cancer, 45 years; positive family history (endometrial cancer, 50 years; colorectal cancer, 60 years) (99)	No frequency	6 homozygous cases with central nervous tumours and/or haematological tumours before 10 years (39, 57)	Frameshift (cryptic exonic acceptor site): r.2007_2023del (p.Ser669Argfs*9) In-frame exon skipping: r.2007_2174del (p.Ser669_Ala725delinsArg) (39)
c.2117del	Frameshift	Colorectal cancer, 51 years; positive family history (colorectal cancer, 37 and 51 years; breast cancer, 45 years) (99) Colorectal cancer, 53 years (8) Colorectal cancer, 53 years; positive family history (CO (colorectal cancer 43 and 70 years; uterine cancer, 50 years) (100) Colorectal cancer, 49 years; positive family history (ovarian cancer, 43 years; gastric cancer, 51 and 62 years) (101) Ovarian cancer (102) Colorectal cancer (103)	0.00000825 (exomes), failQC	2 homozygous cases with central nervous tumours before 10 years (51) 1 compound heterozygous case with haematological tumours before 10 years (34, 47)	Founder variant in French-Canadian Quebec population (103)
Late-onset (≥10 years) CMMRD group					
c.1A>G	Startloss	Colorectal cancer (99, 104–106) Uterine/endometrial cancer (107, 108) Renal cancer (105, 109) Breast and/or ovarian cancer (102, 110)	0.00002419	4 compound heterozygous cases with gastro-intestinal tumours (mainly) and central nervous tumours after 10 years (10, 43)	Recurrent variant (111) NMD escaping variant (VEP)
c.2002A>G	Missense	Breast cancer (112)	No frequency	15 homozygous cases with gastro-intestinal tumours (mainly) and central nervous tumours after 10 years (26, 36, 60)	Missense variant that induce mis-splicing, with residual protein function and atenuated CMMRD phenotype (113) The most frequent reported heterozygous PMS2 variant, founder variant in Inuit population, Northern Quebec (60) Hypomorphic variant (114)
c.2458dup	Frameshift		No frequency, low coverage	4 homozygous cases with central nervous tumours after 10 years (45)	NMD escaping variant (VEP)
c.2531C>A	Missense		No frequency, low coverage	3 homozygous cases with gastro-intestinal tumours (mainly) and central nervous tumours after 10 years (28, 57)	Hypomorphic variant, late onset CMMRD, mimicking LS (114)

### CMMRD-related *PMS2* variants are more frequent than controls in gnomAD v4

**FIGURE 5:**
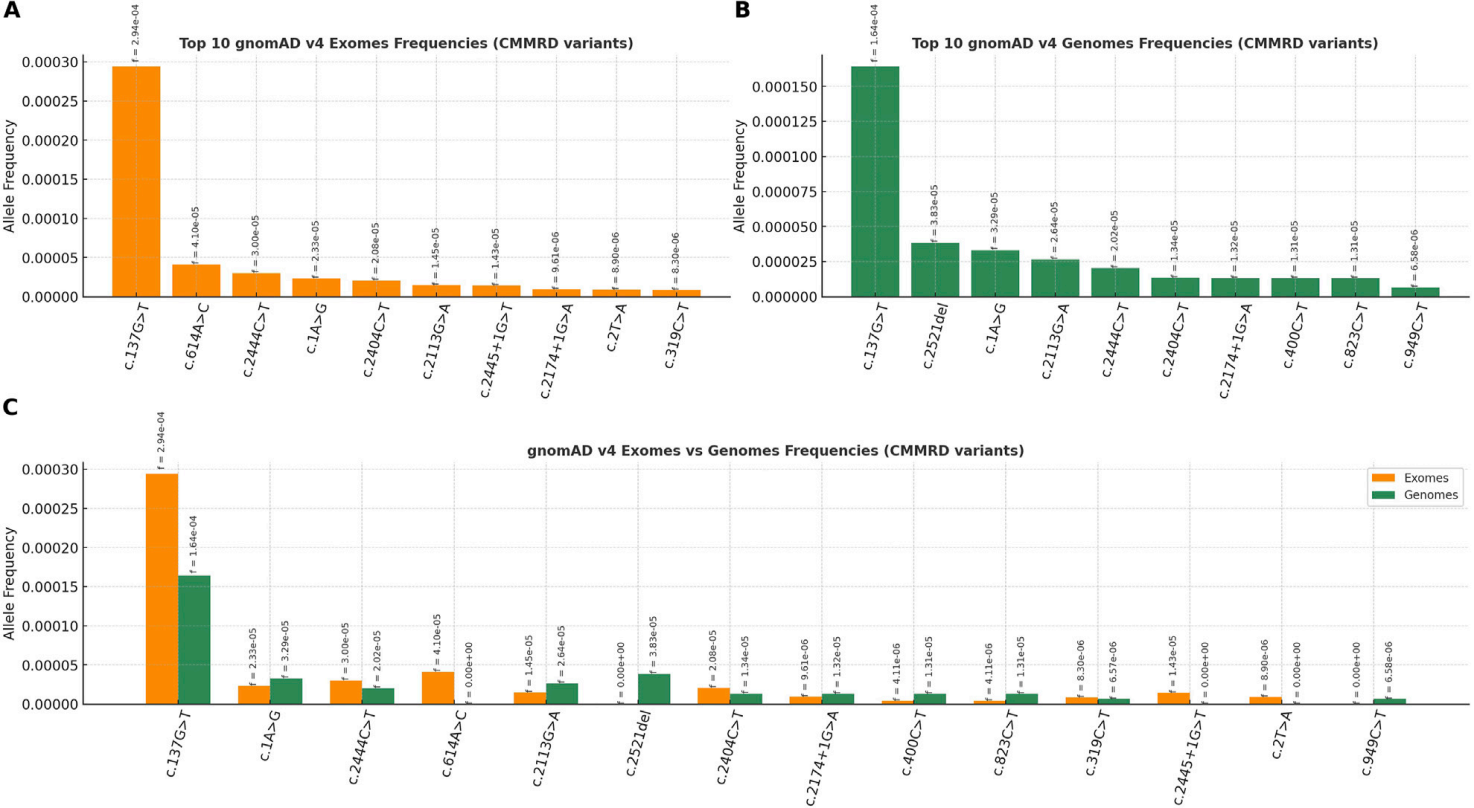
The most frequent *PMS2*-CMMRD variants in healthy population (gnomAD v4), ranked by allelic frequency: **(A)** gnomAD exomes, **(B)** gnomAD genomes and **(C)** gnomAD exomes and genomes (combined).

Out of 74 variants identified in CMMRD cases, the paralogous specific variant (PSV) c.2186_2187del was, as expected, very frequent in the gnomAD v4 dataset (115). The remaining 73 CMMRD-associated variants were more frequent in the general (presumably healthy) population compared to the control group of 733 *PMS2* variants (p < 0.001, Mann–Whitney U test). The most prevalent CMMRD variants in gnomAD v4 are illustrated in Figure 5.

### The combined VarChat–VarSome strategy is the most effective method for variant detection in literature

**FIGURE 6:**
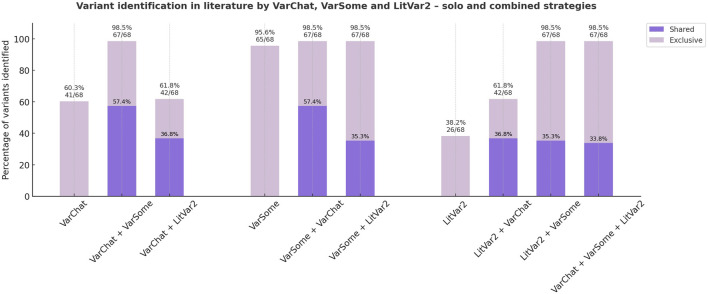
Literature discovery strategies for previously reported *PMS2*-CMMRD short variants (ClinVar). Both individual and combined approaches to literature review are illustrated. Variants identified by at least two individual tools are highlighted in dark purple, while those detected by only one tool are shown in light purple.

All 102 CMMRD-related *PMS2* variants included in ClinVar were investigated using three complementary approaches (VarChat, VarSome and LitVar2) to find supporting literature. A combined search employing both VarChat and VarSome identified PubMed indexed publications for 67/68 (98.5%) confirmed CMMRD-related *PMS2* variants, outperforming each individual tool (Figure 6). None of the tools were able to find relevant literature for the *PMS2*, c.2458dup CMMRD variant, making it the only variant missed by the combined approach.

Of the 102 variants analysed, 13 (12.74%) were located in the same canonical splice sites as other *PMS2* splicing variants previously reported in the literature but lacked direct evidence supporting their role in CMMRD. Similarly, 5/102 variants (4.90%) without supporting literature affected the start codon. Two frameshift and nonsense variants, c.320_321insT and c.543T>G, had the same predicted protein consequence as other confirmed CMMRD variants. One false positive, c.1376C>A, was erroneously reported by testing laboratories in a CMMRD case (Variation ID: 135936, Accession: SCV005474595.1), where the actual variant was c.1376C>G (93). Another variant, c.1731_1732delinsAGT, was misreported (Variation ID: 9244, Accession: SCV001173401.5), possibly due to a nomenclature-related issue regarding c.1730dup variant, which was described as c.1730_1731insA in the original publication (85).

## Discussion

Our study provides a comprehensive review of reported cases of constitutional mismatch repair deficiency (CMMRD) associated with *PMS2* pathogenic and likely pathogenic variants submitted to ClinVar by diagnostic and research laboratories. CMMRD is a rare autosomal recessive cancer predisposition syndrome, characterised by early onset of malignancies during childhood, most commonly involving central nervous system, gastro-intestinal tract and hematopoietic system (1–3). By analysing 133 cases and families with *PMS2*-associated CMMRD, we confirmed this characteristic pattern of tumor predisposition observed in the syndrome. Brain tumors occurred earliest in the course of disease, most frequently followed by gastro-intestinal malignancies. However, in line with the literature, we also identified a subset of cases with later-onset disease, often associated with previously recognized hypomorphic *PMS2* variants, as well as new candidate variants predicted to have a mild impact on protein function (60,114). Splicing variants predicted to cause in-frame exon skipping or affecting exons with constitutive leaky splicing were enriched in cases of late-onset neoplasias. Several of these variants involved exons 4, 6, 8 and 10, which our group previously identified as being prone to exon skipping (116). These cases commonly presented with gastro-intestinal tumors as the first malignancy. Moreover, a significant proportion of the analysed cases presented with cutaneous findings, mainly café-au-lait macules and freckles, which are known features shared with other rare genetic disorders such as neurofibromatosis type 1 and Legius syndrome. In this context, our study reinforces the importance of early recognition of these clinical features and timely molecular diagnosis in distinguishing CMMRD from phenocopies, thereby ensuring precise clinical management and appropriate genetic counselling (4–6).

Two truncating *PMS2* variants, c.2007-2A>G and c.2117del, were exclusively associated with a highly penetrant phenotype in our cohort, with brain tumors as the main clinical presentation occurring before the age of 10. However, these findings provide only limited information regarding the penetrance of these variants in heterozygous state. *PMS2* is generally considered a low to moderate penetrance cancer predisposition gene, typically associated with milder forms of Lynch syndrome (8,10–12). Moreover, many pathogenic *PMS2* variants are expected not to manifest any cancer phenotype over the course of individual’s lifetime (8). In LS families related to both c.2007-2A>G and c.2117del *PMS2* variants, a high penetrance of cancer phenotype across several generations could be observed, with neoplasia mostly occurring after the age of 40 (99–101). For c.2007-2A>G, calculated constitutional microsatellite instability (cMSI) score in blood of homozygous individuals with CMMRD showed a significantly increased value compared to other *PMS2* variants, indicating a more severe phenotype (117). Whether c.2007-2A>G and c.2117del truly confer a higher penetrance compared to other *PMS2* variants remains unclear and represents an important topic for future research.

Moreover, the *PMS2* variants under analysis were significantly more frequent in the healthy population, which might be indicative of a role for lower-penetrance variants in the pathogenesis of CMMRD. Consistent with this observation, an enrichment of variants with milder predicted functional impact was noted among cases where the first neoplasia occurred after the age of 10. Further studies on larger datasets are needed to determine whether lower-penetrance *PMS2* variants must be coinherited with another pathogenic allele to cause CMMRD, similar to the genetic model recently described in Fanconi anemia (FA) (15), in which one *BRCA1* or *BRCA2* variant retains partial protein function to ensure embryonic viability. Additionally, it remains unclear whether variants exclusively associated with early and later-onset CMMRD significantly differ in penetrance, an important distinction to be made, with potential clinical implications for the management of individuals with *PMS2-*LS.

### Limitations

This systematic review was performed retrospectively, with all recognized limitations for this specific type of study, including potential selection and reporting biases. The current study did not include CMMRD cases for which causative variants were not reported in ClinVar. However, this is not expected to significantly alter the main observations regarding disease phenotype. The literature review relied predominantly on semi-automated tools, which could have influenced the detection or inclusion of some relevant cases. Nonetheless, this approach enabled the development of a combined literature review method, that allows a faster and more scalable data extraction. Moreover, our findings underscore that manual literature review remains essential following the initial screening process to ensure the accuracy and reliability of the information. In the reviewed cases, molecular diagnosis was primarily established through targeted *PMS2* gene testing, as a direct consequence to the known issue of gene–pseudogene interference in clinical practice (69,72,118–120). This strategy, however, does not allow for identification of other genetic modifiers that may modulate the phenotype. Tools based on low-pass genome sequencing, such as LOGIC, aim to address this gap (121,122). Although the MMRDness score shows correlations with age at onset and severity of the phenotype, this method falls short in detecting other genomic variants that may account for phenotypic variability among cases. Future research involving larger datasets and prospective cohort studies, using whole genome sequencing as the testing method would be valuable for further expanding and validating genotype–phenotype correlations.

## Conclusion

In summary, *PMS2*-associated constitutional mismatch repair deficiency (*PMS2*-CMMRD) represents a heterogenous clinical and genetic entity. Our study highlights emerging genotype–phenotype correlations in *PMS2*-associated CMMRD, which may contribute to refining prognosis and guiding clinical care in both CMMRD and LS. Notably, we identified several candidate *PMS2* variants that represent promising targets for future penetrance studies. Larger datasets and prospective cohort studies are warranted to further validate these observations.

## Data Availability

The raw data supporting the conclusions of this article will be made available by the authors, without undue reservation.

## Abbreviations

BRCA1, Breast Cancer 1, Early Onset; BRCA2, Breast Cancer 2, Early Onset; CMMRD, Constitutional Mismatch Repair Deficiency; CNS, Central Nervous System; cMSI, Constitutional Microsatellite Instability; DNA, Deoxyribonucleic Acid; GI, Gastrointestinal; IHC, Immunohistochemistry; LS, Lynch Syndrome; MLH1, MutL Homolog 1, Mismatch Repair Protein; MLPA, Multiplex Ligation-dependent Probe Amplification; MMR, Mismatch Repair (inferred from context, commonly paired with MMRDness); MSH2, MutS Homolog 2, Mismatch Repair Protein; MSH6, MutS Homolog 6, Mismatch Repair Protein; NGS, Next-Generation Sequencing; NF1, Neurofibromin 1; PCR, Polymerase Chain Reaction; PMS2, PMS1 Homolog 2, Mismatch Repair Protein; RNA, Ribonucleic Acid; RT-PCR, Reverse Transcription Polymerase Chain Reaction; VEP, Variant Effect Predictor.
